# Evaluation of a rapid immunochromatographic test kit to the gold standard fluorescent antibody test for diagnosis of rabies in animals in Bhutan

**DOI:** 10.1186/s12917-020-02405-4

**Published:** 2020-06-08

**Authors:** Tenzin Tenzin, Kelzang Lhamo, Purna B. Rai, Dawa Tshering, Pema Jamtsho, Jamyang Namgyal, Thrinang Wangdi, Sangay Letho, Tuku Rai, Sonam Jamtsho, Chendu Dorji, Sangay Rinchen, Lungten Lungten, Karma Wangmo, Lungten Lungten, Pema Wangchuk, Tshewang Gempo, Kezang Jigme, Karma Phuntshok, Tenzinla Tenzinla, Ratna B. Gurung, Kinzang Dukpa

**Affiliations:** 1 Department of Livestock, National Centre for Animal Health, Serbithang, Thimphu, Bhutan; 2 District Veterinary Hospital, Trashigang, Bhutan; 3 Regional Livestock Development Centre, Kanglung, Trashigang, Bhutan; 4 City Veterinary Hospital and Satellite Veterinary Laboratory, Phuentsholing, Bhutan; 5 Regional Livestock Development Centre, Tshimasham, Chukha, Bhutan; 6 Satellite Veterinary Laboratory, Deothang, Samdrup Jongkhar, Bhutan; 7 District Veterinary Hospital, Samtse, Bhutan; 8 Regional Livestock Development Centre, Zhemgang, Bhutan; 9 Satellite Veterinary Laboratory, Gelephu, Sarpang, Bhutan; 10 District Veterinary Hospital, Samdrup Jongkhar, Bhutan

**Keywords:** Rabies virus, Diagnostic test, Fluorescent antibody test, Rapid anigen test, Rapid immunochromatographic test, Bhutan

## Abstract

**Background:**

Rabies kills approximately 59,000 people each year worldwide. Rapid and accurate diagnosis of rabies is important for instituting rapid containment measures and for advising the exposed people for postexposure treatment. The application of a rapid diagnostic tests in the field can greatly enhance disease surveillance and diagnostic activities, especially in resource poor settings. In this study, a total of 179 brain tissue samples collected from different rabies suspect animal species (113 dogs, 50 cattle, 10 cats, 3 goats, 2 horses, and 1 bear) were selected and tested using both rapid immunochromatographic kit and the reference standard fluorescent antibody test (FAT). We evaluated the sensitivity, specificity, positive predictive value (PPV) and negative predictive value (NPV) of a rapid antigen detection test kit produced by BioNote, Inc. (Hwaseong-si, Korea) relative to a FAT for its fit-for-purpose for confirmation of clinical cases of rabies for early response and enhancing rabies surveillance.

**Results:**

Among 179 samples examined in this study, there was a concordance in results by the rapid test and FAT in 115 positive samples and 54 negative samples. Test results were discordant in 10 samples which were positive by FAT, but negative (false negative) by rapid kit. The rapid test kit showed a sensitivity of 92% (95% CI: 85.9–95.6) and specificity of 100% (95% CI: 93.4–100) using FAT as the reference standard. The positive and negative predictive values were found to be 100% (95% CI:96.7–100) and 84.4% (95% CI: 73.6–91.3), respectively. Overall, there was 94.4% (95% CI: 90–96.9) test agreement between rapid test and FAT (Kappa value = 0.874) with a positive percent agreement and negative percent agreement of 92 and 100%, respectively.

**Conclusions:**

Our finding demonstrated that the rapid test kit (BioNote) can be used for rabies surveillance and confirming clinical case of rabies in animals for making rapid decisions particularly controlling rabies outbreaks in resource poor settings.

## Background

Rabies is a 100 percent fatal zoonotic disease caused by *Lyssavirus* of the family *Rhabdoviridae* and transmitted mainly through the bite of a rabid animal. However, canine rabies is preventable through three proven and effective interventions – awareness education (i.e. making people aware of how to avoid the bites of rabid dogs and to seek treatment when bitten), mass dog vaccination (i.e. stopping the transmission of rabies at its source when a sufficiently high proportion of the animal reservoir population is immunized), and post-exposure treatment (PET) including prophylaxis and wound care. Despite the availability of these tools, around 59,000 people die from the disease each year worldwide [1]. Approximately 80% of human cases occur in rural areas of an economically disadvantaged countries in Africa and Asia, and over 40% of rabies deaths occur in children aged less than 15 years [1]. Dogs are responsible for the transmissions of up to 99% of human rabies cases in the world. Therefore, rapid and accurate diagnosis of rabies following exposure to a rabies suspect animal is important for instituting rapid containment measures in animals and for advising the exposed people for postexposure treatment.

**Fig. 1:**
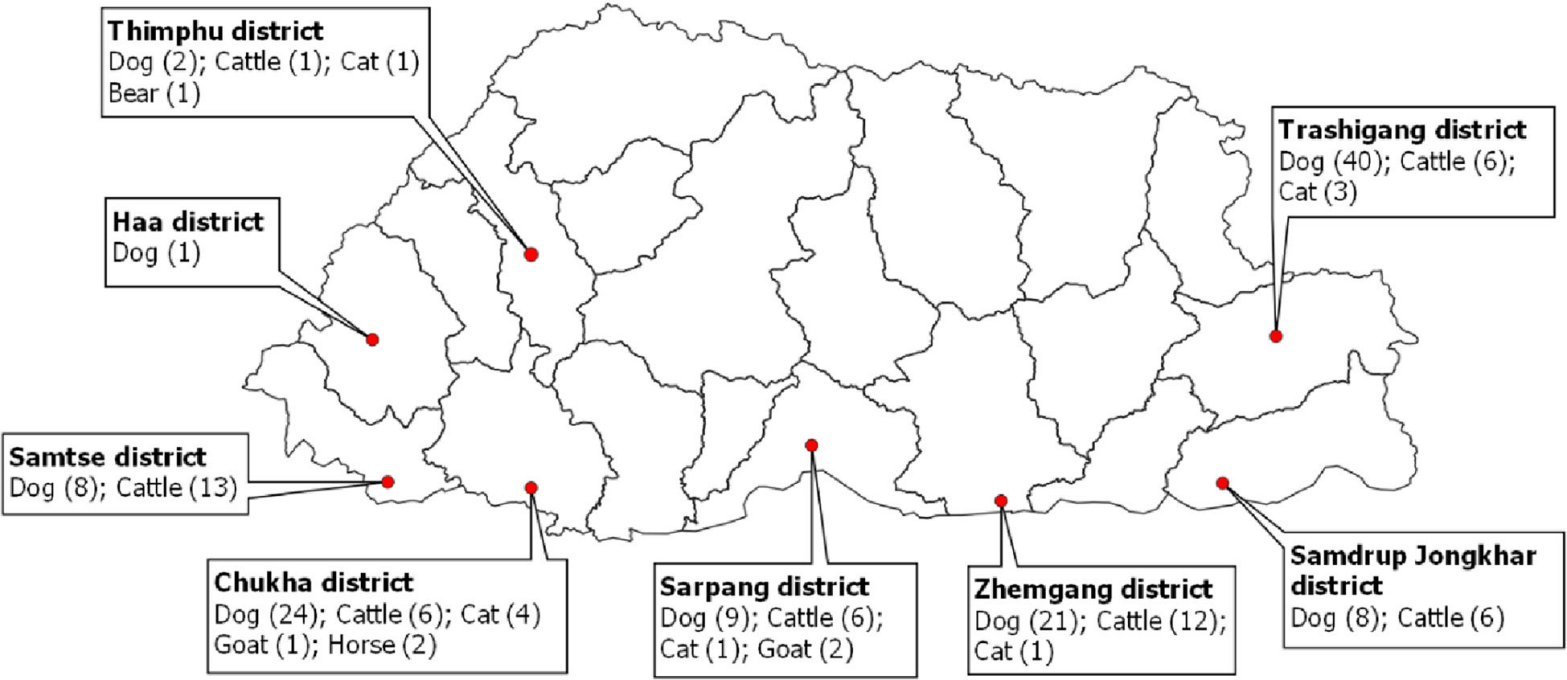
District map of Bhutan showing the origin of brain tissue samples from different species of animals between 2012 and 2017. The number within the bracket against different animal is the total number of brain tissue samples collected and tested using both rapid test and fluorescence antibody test. Excepting Thimphu and Haa, other districts where the samples were collected from are endemic for rabies while Trashigang in the east report sporadic cross-border outbreaks of rabies. The map was prepared using Quantum GIS, QGIS Development Team (2019), QGIS Geographic Information System, Open Source Geospatial Foundation Project (http://qgis.osgeo.org) and was not taken from another source

In Bhutan, rabies in animals are prevalent mostly in the southern parts of the country that shares border with India (Fig. 1) [2, 3]. Sporadic outbreaks are also reported in the interior rabies-free areas, especially in eastern Bhutan as a result of incursions and spread from the border areas [4–6]. Between 2006 and 2016, 17 humans have died of rabies in Bhutan, the majority being children below 15 years of age and around 7,000 dog bite cases (1026 bites per 100,000 people annually) in humans are reported every year in the country [7–9]. Nevertheless, Bhutan is on the right track to achieve zero human deaths from dog-mediated rabies by 2030 as several control measures are in place in line with the global strategic plan of “Zero by 30” [3, 10, 11].

The surveillance of rabies depends on investigations conducted on animals showing clinical signs suggestive of rabies, history of exposure and on laboratory investigations of brain tissue samples from carcasses. In Bhutan, the rabies suspect cases in animals are diagnosed based on the clinical signs such as change in behavior progressing to aggression, biting people or animals, excessive salivation, change in bark tone, paralysis, coma and death [11]. The clinical cases are confirmed by testing brain tissue samples using immunochromatographic test commonly called as rapid anigen antigen detection test kit produced by BioNote, Inc. (Hwaseong-si, Korea) [12] in the field and the negative and inconclusive results are further confirmed by fluorescent antibody test (FAT) [13]. The Anigen Rapid Rabies Ag Test Kit is a chromatographic immunoassay (lateral flow assay) for the qualitative detection of rabies virus antigen in canine, bovine, raccoon dog’s secretions of saliva, and brain homogenates. Since 2012, the rapid kit produced by BioNote, Inc. (Hwaseong-si, Korea) has been used in Bhutan for field diagnosis of rabies and has enhanced rabies surveillance and rapid response to outbreak in the country [3, 14]. Since it is expensive to set up FAT or molecular diagnostic facilities in all rabies endemic areas in the country, a rapid, accurate, inexpensive and easy to perform test is necessary to diagnose rabies in the field so that immediate control measures can be taken to prevent spread of the outbreak and save human lives.

The aim of this study was to evaluate the test sensitivity, specificity, positive predictive value (PPV) and negative predictive value (NPV) of a rapid antigen detection test kit produced by BioNote, Korea relative to a FAT for clinical diagnosis of rabies virus in various species of domestic and wild animals in Bhutan.

## Results

Among 179 samples data examined in this study, there was concordance in results by the rapid test and FAT in 115 positive samples and 54 negative samples. Test results were discordant in 10 samples which were positive by FAT, but negative (false negative) by rapid test kit (Table 1). Thus, the rapid antigen detection test kit had sensitivity of 92% (95% CI: 85.9 – 95.6) and specificity of 100% (95% CI: 93.4 – 100) relative to FAT. The positive and negative predictive values were found to be 100% (95% CI: 96.7 – 100) and 84.4% (95% CI: 73.6 – 91.3), respectively. Overall, there was 94.4% (95% CI: 90.0 – 96.9) test agreement between rapid test and FAT (Kappa value = 0.874) with a positive percent agreement of 92% and a negative percent agreement of 100%. The test discrepancies were observed with dog and cattle samples. The rapid test kit tested 45 dog sample negative when compared to only 37 samples being tested negative by FAT (i,e, eight sample were tested as false negative by rapid test). Similarly, rapid test kit tested nine cattle sample negative when compared to only seven samples tested negative by FAT (i,e, two sample were tested as false negative by rapid test) (Table 2). The eight dog samples that produced discordant results were from stray dogs.

**Table 1:** Cross-tabulation of the rapid test results by the results of the fluorescence antibody test (FAT) of brain tissue samples in animals in Bhutan (2012-2017)

	FAT positive	FAT negative	Total
Rapid test positive	115	0	115
Rapid test negative	10	54	64
Total	125	54	179

Sensitivity = 115/125 = 92%; Specificity =54/54 = 100%; Positive Predictive Value = 115/115 = 100%; Negative Predictive Value = 54/64 = 84.4%

**Table 2:** Comparison of rabies diagnosis results obtained with fluorescence antibody test (FAT) and a rapid antigen test kit on 179 species of animals in Bhutan (2012-2017)

Samples	Result	FAT	Rapid test
Bear (*n* = 1)	Positive	0	0
	Negative	1	1
Cat (*n* = 10)	Positive	2	2
	Negative	8	8
Cattle (*n* = 50)	Positive	43	41
	Negative	7	9
Dog (*n* = 113)	Positive	76	68
	Negative	37	45
Goat (*n* = 3)	Positive	3	3
	Negative	0	0
Horse (*n* = 2)	Positive	1	1
	Negative	1	1
**Total (*n*** **= 179)**	**Positive**	**125**	**115**
	**Negative**	**54**	**64**

## Discussion

The use of rapid diagnostic tests in the field can greatly enhance disease surveillance, confirm clinical case and facilitate rapid response activities, especially in resource poor settings. In the present study we have evaluated a commercially available rabies rapid kit produced by BioNote, Inc. (Hwaseong-si, Korea) for its fit-for-purpose for confirmation of clinical cases of rabies in animals for early response and enhancing rabies surveillance as recommended by OIE [13]. We first screened all suspected rabies brain samples using the rapid test in the field and then re-confirmed by the FAT. The results showed that the rapid test is highly sensitive and specific which agrees with findings from other studies conducted elsewhere (15-21). Other studies have also evaluated the rapid kit BioNote, Inc. (Hwaseong-si, Korea) relative to the FAT for detecting rabies virus in the brain samples and have reported diagnostic sensitivities either higher (95 to 100%) [15–21] or lower (88.3 to 91.7%) [22–24] while most of the diagnostic specificities were also between 98.9 to 100% [18]. However, two studies [25, 26] detected poor sensitivity of the existing commercially available rapid kits when compared with FAT and the molecular test, but the sensitivity of rapid kit (BioNote) was higher (62-100%) than the other commercially available rapid test kits. The Rapid kit produced by the BioNote target the N-gene (nucleoprotein) while other kits do not have information on which antigen it is targeted and thus may be related to poor sensitivities [22, 26].

Our study detected false negative results in 5.6% (10 of 179) of the samples relative to FAT. This could have been due to the low viral load in the samples that was not detected by the rapid kit as compared with the FAT [26] since the rapid kit has low analytical sensitivity to be able to detect only about 102.0LD50/0.03ml in mice in both brain and saliva samples [12]. It has been observed that specimens with a high antigen load were more likely to also test positive with the BioNote kit test than the ones with a low antigen load [26]. For instance, eight of the ten discordant samples that tested negative to rapid test and positive by FAT were from stray dogs that were either killed by the public or euthanized during outbreak response. In most instances, stray dogs that demonstrates clinical signs suggestive of rabies are killed by the public. Possibly the stray dogs were destroyed during the early disease stage, thus resulting in lower concentration of virus load in the brain. Therefore, confirmatory tests are recommended in cases of inconclusive and negative results. The human factors such as lack of experience in sampling and performing the tests could also have been responsible for the variable results. Most of the studies conducted elsewhere had evaluated the rapid test with FAT and other tests in the laboratory setting where the samples were collected from the field and shipped to the laboratory for analysis, and possibly the tests were also performed by a single trained technicians for all the samples thus enhancing the repeatability and reproducibility of the test, thereby reducing the incidence of false results. In our study, the disease investigation team comprising of the veterinarians and the laboratory technicians visited the outbreak area or suspect cases, collected the fresh brain tissues from carcasses and in most cases performed test in the field (on site of death of animals) providing immediate results to the community or the owners for control measures. Therefore, variation of rapid test results in our study could be due to the varying capacity of the field veterinarians and technicians. Our rapid test results could have been improved if the same technician performed all the test and in a laboratory setting, but this was not logistically feasible. Most importantly this rapid test is meant to be used in the field in resource poor setting that can provide immediate results. Moreover, we did not perform rapid test for the second time at the national laboratory for all the samples other than FAT to reconfirm the rapid test results submitted from the field to avoid wastage of the limited kits. Even at the world rabies reference laboratory setting, substantial variation between the different laboratories were observed with the test sensitivity ranging from 33 to 100% of the rapid kit (BioNote) when compared with FAT and RT-qPCR [26].

In Bhutan, FAT is the only reference standard test available for diagnosis of rabies and is a routine diagnostic test for rabies recommended both by WHO and OIE [13]. Although FAT is highly sensitive and specific (between 98% and 100%) it depends heavily on the quality of the immunofluorescent conjugate, the maintenance of the fluorescent microscope and on an experienced technician performing test and reading the microscope slides [13]. Sensitivity also depends on the specimen, the degree of autolysis and the sample type [13]. In our study, we evaluated the rapid kit (BioNote) relative to a FAT to confirm clinical case of rabies in animals for implementing early response in the field and enhance rabies surveillance. Although the positive test results of the rapid test indicate positive to rabies virus and were consistently positive using FAT, the negative test results, however, were interpreted cautiously and were always correlated with the history of bite, clinical signs of rabies and were further confirmed by FAT. Thus, no fatal outcome in human was reported due to false negative results of the rapid test in Bhutan. Like in our study, many studies [18, 26] have reported high specificity (98.9 to 100%) of the rapid test (BioNote), so the false positive outcomes were low which would otherwise may lead to unnecessary use of limited resources including PEP and response activities [26]. However, to our knowledge, only one study has reported false positive results (two false positive among 30 FAT negative specimens), with a specificity of 93.3% [16].

It is to be noted that apart from various commercially available rapid test kits, there are other rapid, inexpensive and field-friendly assays, such as the direct rapid immunohistochemical test (dRIT) which is recommended by OIE as an alternative to FAT in routine rabies diagnosis [13, 27, 28]. This test has similar sensitivity and specificity as in FAT and the test can be used in laboratories that do not have access to a fluorescence microscope [27, 28].

In Bhutan, lack of point-of-care tests in the past delayed the implementation of rapid response to suspected rabies outbreaks since samples had to be transported for days to the national laboratory for confirmation even though the preemptive control measures were implemented based on clinical signs and epidemiological findings. Following the introduction of this rapid kit in the country in 2012, the epidemiologic surveillance and diagnosis of rabies in remote areas have greatly improved. The use of rabies rapid tests complemented with epidemiological findings have enhanced the capacity of the field veterinary centres to respond promptly to any suspected outbreaks of rabies by vaccinating dogs against rabies and preventing spread of disease outbreaks. It had also facilitated in making immediate decision to provide post-exposure prophylaxis in human, thus saving human lives from rabies deaths [29].

The main advantage of the rapid test kit is that it is inexpensive, rapid and easy to use in the field or in laboratory setting without the need for a microscope, electricity and yield results within 5-10 minutes. Our data, along with those from many previous studies, also support the use of rapid test for rabies clinical diagnosis of rabies and surveillance in regions where other standard diagnostic facilities cannot be practically implemented. In resource-constrained countries such as Bhutan, such rapid kits have greatly enhanced the possibility of providing rapid response to suspected rabies cases and reduced further spread of rabies both in animals and humans. As Bhutan is close to eliminating dog-mediated human rabies, enhanced and sensitive surveillance is a prerequisite to rapidly detect and eliminate rabies virus circulation in animal populations.

## Conclusions

Our finding demonstrated that the rapid test kit (BioNote) can be used for enhancing rabies surveillance and confirming clinical case of rabies in animals for making rapid decisions, particularly controlling rabies outbreaks in resource poor settings.

## Methods

### Description of sample collection

Rabies is a notifiable disease in Bhutan and any suspected cases must be reported to the veterinary authority for investigation [11]. Following report of any rabies suspect case(s) in animals in the community, veterinarian and or laboratory technician (listed authors in this paper) conducted investigation and collected brain tissue samples (the brain stem, hippocampus and cerebellum) by opening the skull in their respective region. Protective personal equipment (such as hand gloves, face mask and eye google) were always worn while opening the skull and collecting the brain samples. The team also received pre-exposure prophylaxis against rabies. Most of the rabies suspect stray dogs would have been already killed by the public when the team arrived at the site for investigation and sample collection while some suspect cases were euthanized to collect the samples to confirm the case. However, the samples from other livestock were collected after the death of animals since it was culturally sensitive to euthanize the animals. The diagnostic samples in this study included both from carcasses and euthanized animals. Majority of the samples had originated from rabies endemic areas in the southern part of Bhutan that share border with India (Fig. 1) while few samples were also collected from rabies-free interior Bhutan and tested as part of the routine surveillance program.

Firstly, a rapid test was performed in the field using fresh brain tissue (described below) and the second batch of samples were preserved in phosphate glycerol saline (50%) and shipped to the national veterinary laboratory located at the National Centre for Animal Health (NCAH), Thimphu for conducting FAT. Depending on the outbreak location, sample transport from the field to the national laboratory would normally take about 2-5 days.

### Description of rapid immunochromatographic test (Anigen rapid Ag test)

We used Anigen Rapid Rabies Ag Test Kit (BioNote, Inc, Hwaseong-si, Korea) in the field. The basic principle behind this test is the fluid migration of a sample along a nitrocellulose membrane. Gold conjugated antibodies are directed against epitopes of the rabies virus nucleoprotein [22, 26] and the antigen-antibody complex is then immobilized by a second antibody which is fixed on the test strip. The rapid test kit has a letter of “T” and “C” as test line and control line on the surface of the device. Both the test line and control line in result window are not visible before applying any samples. The control line is used for procedural control. Control line should be always appeared if the test procedure is performed properly and the test reagents of control line are working. A purple test line will be visible in the result window if there are enough rabies virus antigen in the specimen. The detection limit of this kit is reportedly 102.0LD50/0.03ml in mice [12].

**Fig. 2:**
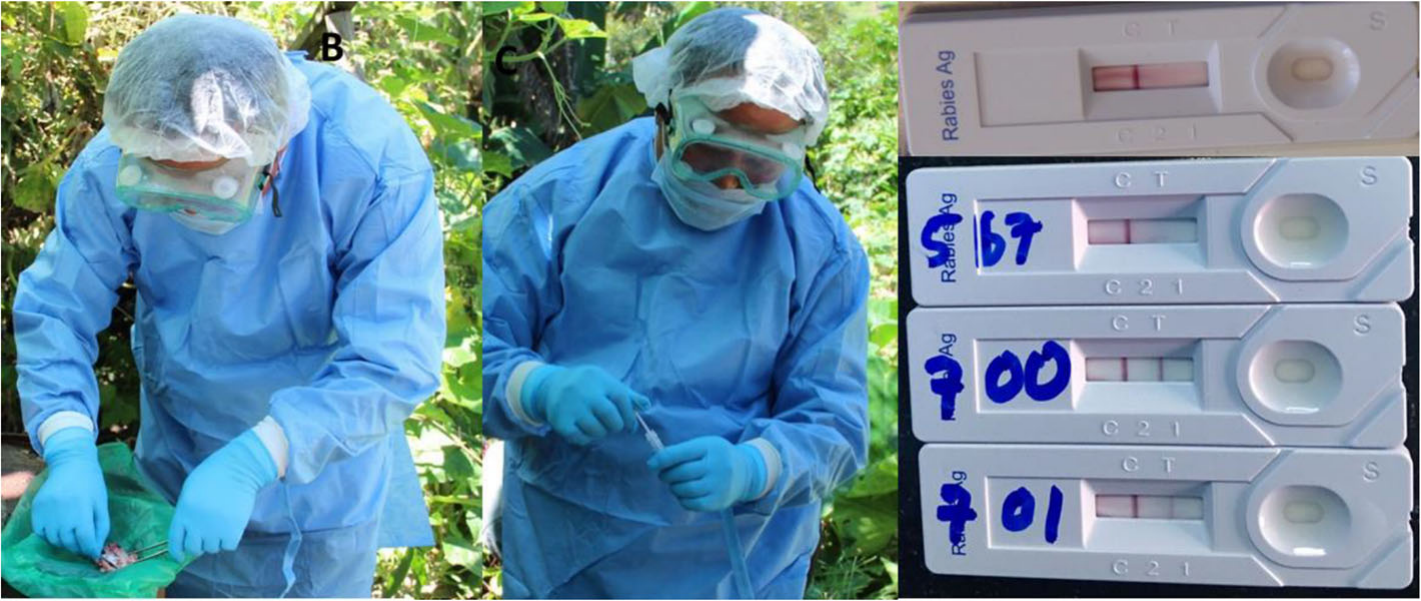
Harvesting brain tissues from a carcass (cat) and performing rapid test in the field (on the site of death). Rapid test positive to rabies virus showing clear “C” and “T” line (bottom 2 test cassette); rapid test negative to rabies virus showing only one line (under “C” line (upper 2 test cassette). The images were captured while performing the rapid test and was not taken from another source

For this study, the rapid test was performed immediately after harvesting the fresh brain tissue samples in the field according to the manufacturer’s instruction (BioNote, Inc) (Fig. 2). Briefly, the cotton swab supplied along with the kit was used to swab the brain tissue (the brain stem, hippocampus and cerebellum) and then dipped the swab into the specimen tube containing 1ml of assay diluent, and stirred/mixed well to ensure a good sample extraction. The swabbing of the brain tissue with swab and mixing into the assay diluent was repeated for 3-5 times to ensure a good sample extraction. The test cassette/device was removed from the foil pouch and placed it horizontally on a flat and dry surface. Using the disposable dropper provided with the kit, four drops of the extracted sample (100μL) was added into the sample hole in the cassette and the result was interpreted within 5-10 minutes according to the manufacturer’s instruction. As the test begin to work, the appearance of two colour bands - one on the control line (C) and the other on test line (T) within the result window, no matter which band appears first indicates a positive result. The appearance of only one colour band within the result window indicates a negative result and if the purple color band is not visible within the result window after performing the test, the result is considered invalid. The test results were immediately shared with the animal owners or any people that had linked with the exposure to visit the hospital for medical advice. All samples whether positive or negative to rapid test were submitted to the national laboratory for performing FAT (described below) to evaluate the test performance in this study. At the national laboratory, the sample details and the rapid test results were recorded in the sample receipt register. Only few samples were retested using rapid test at the national laboratory.

### Description of fluorescent antibody test

**Fig. 3:**
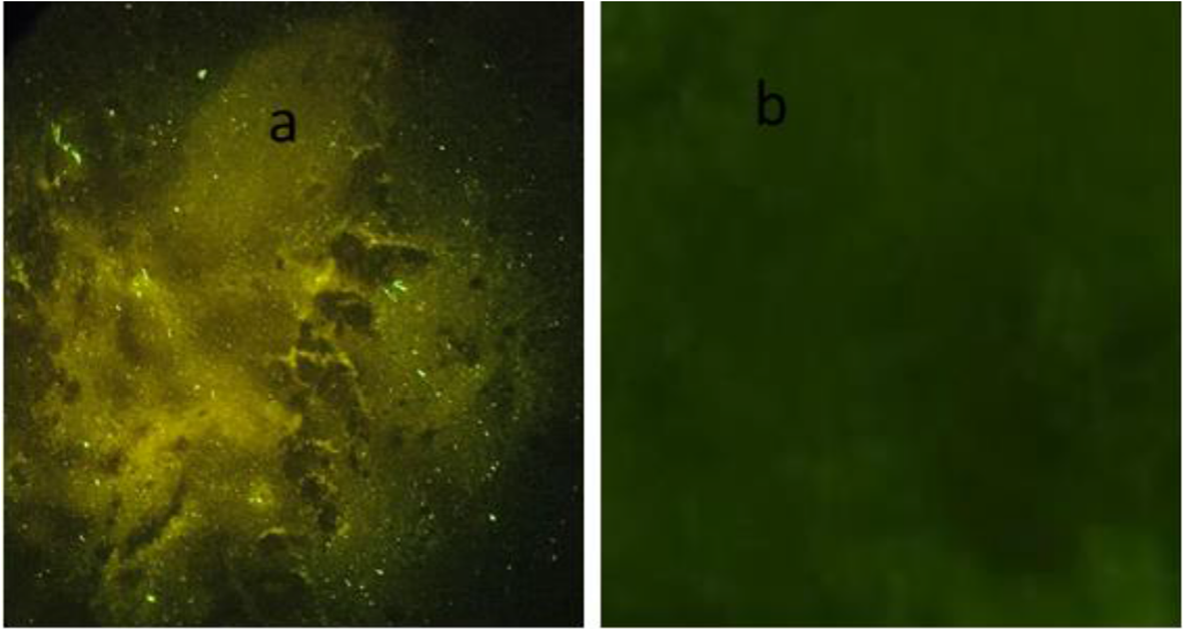
Test results of a fluorescence antibody test demonstrating presence of an apple green fluorescence in brain tissue sample of a dog preserved in 50% glycerol saline indicating positive to rabies virus (**a**) and a brain tissue sample of a dog that tested negative to rabies virus (**b**). The image was captured while reading the test slides and was not taken from another source

The FAT was conducted at the national veterinary laboratory with little modification of the recommended protocol [13]. Briefly, the brain tissue preserved in 50% glycerol saline were washed with buffered saline to remove the glycerol. The brain tissue impression smears were prepared from the composite of the brain stem, cerebellum and hippocampus, air dried and were fixed in chilled acetone for 15-30 minutes. The impression smear slides were air dried and 150 μl of fluorescein isothiocyanate (FITC) conjugate anti-rabies antibody (LIGHT DIAGNOSTICS Rabies DFA Reagent, USA) which was prepared by diluting at 1:20 ratio with PBS (PH 7.4) was added on smear and incubated at 37°C for 30-45 min in humidified dark chamber. The slides were then washed with PBS thrice for 5 min each which will washed away the unbound antibody and then washed with distilled water to stop the reaction. Both positive and negative control brain tissue samples smear were also prepared and stained as in the test samples. After washing, slides were air-dried and mounted in 10-20 % glycerine buffer (pH 7.4) or Faramount, Aqueous Mounting Medium, Ready-to-Use from Dako (Dako North America, Inc, Carpinteria, California, USA) and covered with coverslip. First the positive and negative control slides were examined under fluorescent microscope at 20X which was followed by the test smear samples. The presence of apple green fluorescence was considered as positive results (Fig. 3). The FAT result was then communicated to the field veterinary officials via e-mail and through telephone which were further communicated to the animal owners or people that had linked with exposure and with the human hospital. If the test is negative, the PEP is normally discontinued.

### Data source in this study

**Fig. 4:**
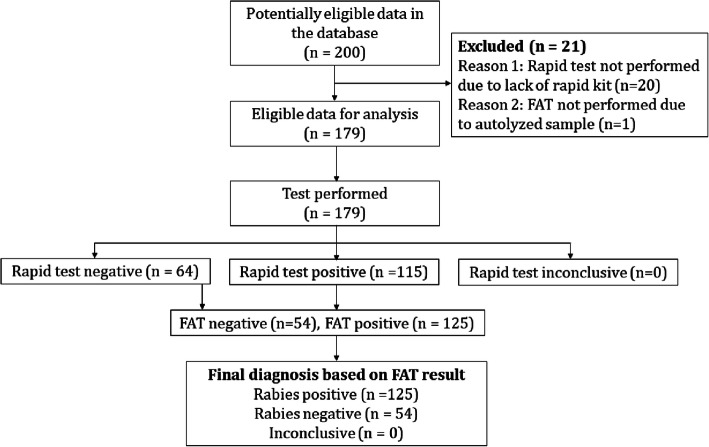
Flowchart showing test results of rapid test kit and fluorescence antibody test (FAT). The flow chart was prepared by following the STARD 2015: An Updated List of Essential Items for Reporting Diagnostic Accuracy Studies (https://www.equator-network.org/reporting-guidelines/stard/) and was not taken from another source

Both the rapid test and FAT results status are maintained at the national laboratory register against the unique sample ID. We retrieved the test results data from the database for the period between 2012 and 2017. The data that contain both rapid test and FAT test results status were included for analysis. Any data that contain only FAT results but not rapid test result status or vice versa were excluded for analysis. Therefore, a total of 179 brain tissue samples data recorded between 2012-2017 from different species of animals (113 dogs, 50 cattle, 10 cats, 3 goats, 2 horses, and 1 bear) met the criteria for the analysis (Fig. 4, Table 3).

**Table 3:** Total number of brain tissue samples collected and tested using both rapid test and fluorescence antibody test (FAT) in animals between 2012 and 2017

Year	Number of samples (percent)
2012	6 (3.35)
2013	25 (13.97)
2014	14 (7.82)
2015	9 (5.03)
2016	36 (20.11)
2017	89 (49.72)
Total	179 (100)

### Data analysis

The data management and analysis were done in Microsoft excel 2010 (Redmond Microsoft, USA) and Stata version 14.0 (Stata Corp, USA). A two by two table was constructed to calculate the test sensitivity, specificity, positive predictive value, negative predictive value of the rapid Ag detection test relative to the FAT. The confidence intervals for the sensitivity and specificity were calculated by using the exact binomial distribution.

The overall test agreement between the rapid Ag and FAT was calculated using Kappa test where Kappa measure agreement between the tests. The Kappa value was interpreted as: below 0.0 poor agreement; 0.00 – 0.20 slight agreement; 0.21 – 0.40 fair agreement; 0.41 – 0.60 moderate agreement; 0.61 – 0.80 substantial agreement and 0.81 – 1.00 almost perfect agreement [30]. A pair of agreement measures, positive percent agreement (PPA) and negative percent agreement (NPA) which are analogous to a sensitivity and specificity calculations were also calculated to capture the difference in agreement between samples that are positive and negative according to the reference standard (FAT).

## Data Availability

The dataset analyzed in this study are presented in this paper and is also available upon reasonable request from the corresponding author.

## Abbreviations

OIE: World Organisation for Animal Health
WHO: World Health Organisation
FAT: Fluorescence Antibody Test
dRIT: Direct rapid immunohistochemical test
PPV: Positive predictive value
PPA: Positive percent agreement
PEP: Post-exposure prophylaxis
PET: Post-exposure treatment
NPV: Negative predictive value
NPA: Negative percent agreement
FITC: Fluorescein isothiocyanate conjugate
